# Exploring how nurses assess, monitor and manage acute pain for adult critically ill patients in the emergency department: protocol for a mixed methods study

**DOI:** 10.1186/s13049-017-0421-x

**Published:** 2017-08-01

**Authors:** Wayne Varndell, Margaret Fry, Doug Elliott

**Affiliations:** 1 Prince of Wales Hospital Emergency Department, Randwick, NSW 2031 Australia; 2 Faculty of Health, University of Technology Sydney, Ultimo, NSW 2007 Australia; 3 Director Research and Practice Development Nursing and Midwifery Directorate NSLHD, Level 7 Kolling Building, Royal North Shore Hospital, St Leonards, NSW 2065 Australia

**Keywords:** Critically ill, Emergency department, Emergency nursing, Mixed methods, Pain, Protocol

## Abstract

**Background:**

Many critically ill patients experience moderate to severe acute pain that is frequently undetected and/or undertreated. Acute pain in this patient cohort not only derives from their injury and/or illness, but also as a consequence of delivering care whilst stabilising the patient. Emergency nurses are increasingly responsible for the safety and wellbeing of critically ill patients, which includes assessing, monitoring and managing acute pain. How emergency nurses manage acute pain in critically ill adult patients is unknown. The objective of this study is to explore how emergency nurses manage﻿ acute pain in critically ill patients in the Emergency Department.

**Methods:**

In this paper, we provide a detailed description of the methods and protocol for a multiphase sequential mixed methods study, exploring how emergency nurses assess, monitor and manage acute pain in critically ill adult patients. The objective, method, data collection and analysis of each phase are explained. Justification of each method and data integration is described.

**Discussion:**

Synthesis of findings will generate a comprehensive picture of how emergency nurses’ perceive and manage acute pain in critically ill adult patients. The results of this study will form a knowledge base to expand theory and inform research and practice.

## Background

In Australia, the number of critically ill patients managed in the emergency department (ED) is increasing [1]. Between 2011 and 2016, the number of critically ill patients presenting to the ED increased by nearly 60% [2–4], with over a third of patients (39%) needing intubation and mechanical ventilation [5]. Although care of critically ill patients traditionally occurs in intensive care units, emergency staff are increasingly having to manage critically ill mechanically ventilated patients for extended periods of time [6, 7]. Pain management is an essential component of quality care delivery for the critically ill patient. However, as many as 79% of patients experience moderate to severe pain, whilst intubated and mechanically ventilated from both their initial reason for presentation (e.g. trauma) and required treatments [8]. Iatrogenic causes of pain include clinical procedures, physical examination, endotracheal intubation, mechanical ventilation, insertion of central venous catheters and chest drains; all of which commonly occur during resuscitation and stabilisation of a critically ill patient in the ED [9, 10]. Intravenous analgesia is therefore commonly administered to alleviate pain, suffering, adverse physiological and psychological effects [11], unplanned self-extubation, accidental removal of invasive monitoring devices, or injury to staff [7, 10].

Pain is a subjective, complex and multidimensional concept that is broadly described as an unpleasant sensory experience associated with actual or potential tissue damage [12], which can be influenced by psychological and environmental factors in every individual [13]. Thus, the most reliable and valid indicator of pain is the patient’s self-report, yet for critically ill patients, communication of pain intensity is problematic; particularly for those with altered levels of consciousness, endotracheal intubation, requiring sedation, analgesia and potentially paralysing agents [14]. These factors therefore place the critically ill patient at greater risk of inadequate pain detection, assessment and inappropriate management [15]. In the absence of a patient’s ability to self-report pain, clinicians usually rely on observable pain indicators such as facial grimacing, crying and compliance with mechanical ventilation. These observations then form the basis for identification and evaluation of a patient’s pain intensity [16].

International pain management guidelines recommend frequent assessment, monitoring and reassessment, and use of validated instruments [11]. Historically, relief from pain through the provision of analgesia could only be initiated by a physician [17]. The shifting stance from physician-only initiated pain management to nurse-initiated analgesic protocols has significantly improved the timely delivery of care and symptom management of pain for a broad range of conditions in ED [18, 19]. A series of ED studies examining nurse-initiated analgesic protocols has demonstrated that emergency nurses can safely assess, initiate and administer analgesia to a range of patient groups and ambulatory conditions [17, 20–39], including the titration of intravenous opioids [35, 40].

In the resuscitation area of the ED, emergency nurses are increasingly responsible for the safety and wellbeing of critically ill patients, and are optimally placed to assess and initiate pain relief [24, 41]. However, to date, how emergency nurses detect, assess, influence and manage acute pain for critically ill patients is unknown and has led to development of this research protocol. The objective of this mixed methods study is to examine emergency nurses’ perceptions and practices in assessing and managing acute pain in critically ill patients. Specifically, to:

1. explore emergency nurses’ practices relating to the assessment, monitoring and administration of analgesia to critically ill patients in ED;
2. examine care activities and behavioural patterns, actions, processes within the context of acute pain management;
3. identify factors, perceived facilitators, barriers and workplace characteristics that influence emergency nurses’ practice in pain management of critically ill patients; and,
4. explore how emergency nurses influence pain management decisions or act independently with regards to the critically ill patient in the ED.

## Methods/design

### Settings

New South Wales (NSW) has the highest population of all states and territories in Australia. In 2016 there was 7.4 million people living in NSW encompassing an area of around 800,000km^2^ [42]. Of the 186 public EDs in NSW, 25 are situated within major referral hospitals with capabilities to manage a wide range of highly complex emergency and critical care; 10 of which are designated state trauma centres. In 2016, there were 2.7 million ED presentations in NSW, of which critically ill or injured patients with life-threatening conditions, defined as patients triaged category 1 or 2 using the Australasian Triage Scale (ATS), represented over one-third (*n* = 966,560; 34.8%) of all presentations [2]. Of the 334,112 (34.6%) critically ill patients that had presented to NSW EDs, most (*n* = 49,069; 14.7%) had presented to a major referral ED; less than 2% (*n* = 3970; 1.2%) were major trauma related [43].

### Design

**Fig. 1:**
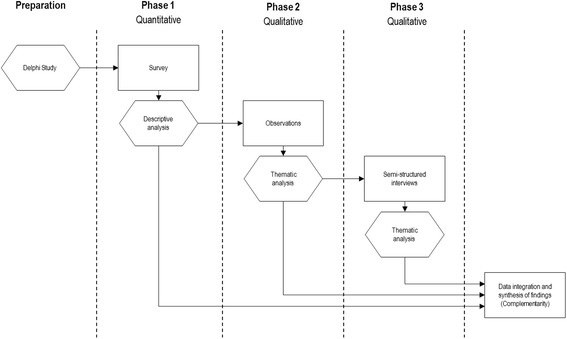
Explanatory sequential mixed methods research design

Emergency healthcare occurs across a complex sequence of complex interactions that are difficult to assess with strictly quantitative or qualitative research methods. Thus, an explanatory sequential mixed-methods (quan → QUAL → QUAL) research design will be conducted in three phases, comprising a survey, observations and semi-structured face-to-face interviews. The study process is outlined in Fig. 1.

To address aims 1, 3 and 4, Phase 1 will use a Delphi technique to develop survey items exploring core outcome variables to measure adequate management of acute pain in critically ill adult patients. The final survey will be administered through a Web-based platform to emergency nurses in NSW. The purpose of the questionnaire is threefold: to enhance the descriptive results of observations and interviews, to validate corresponding observations and statements, and to compare emergency nurses’ perceptions on managing acute pain in critically ill adult patients. Addressing aims 2 and 3, Phase 2 will observe consenting emergency nurses managing critically ill patients in the resuscitation area, specifically behaviours and interactions in detecting, assessing and managing acute pain. To address aims 1, 3 and 4, Phase 3 will interview consenting emergency nurses about their experiences and practices in managing acute pain in critically ill patients. Donabedian’s Quality and Safety model [44], as extended by Coyle and Battles [45], will be used to analyse information obtained in the study. The model provides a framework for examining health services and evaluating quality of health care, including examining clinical practice in healthcare [46], clinician communication [47] and advanced nursing practice [48]. The framework is comprised of four elements: antecedents, structure, process and outcome (Table 1).

**Table 1:** Expanded Donabedian’s quality care framework

Antecedents	Structure	Process	Outcome
Environment	System characteristics Provider characteristics Patient characteristics	Technical style Interpersonal style	Clinical end-points Functional status General well-being Satisfaction with care
Culture			
Social			
Political			
Personal			
Physical			
Health professions			
Patient personal characteristics			

### Sampling, recruitment and consent

#### Survey participants

Recruitment of survey participants will be initially conducted through the College of Emergency Nursing Australasia, which represents over 1400 emergency nurses across Australia. Members of the College will be provided by email and via College social media platforms, information concerning the nature and purpose of the study and a private link to the electronic survey. Nurses working in a NSW ED who have care for critically ill (ATS category 1 or 2) adult (≥16 years old) patients in the last six months will be eligible for participation.

#### Observation participants

Two designated trauma EDs will be randomly selected and approached to seek permission to conduct observations of emergency nurses managing critically ill patients in the resuscitation area. Trauma designated EDs typically manage high volumes of critically ill patients and have the most exposure to managing acute pain in this cohort of patients. Study information will be distributed and discussed at departmental meetings. All emergency nurses able to work in the resuscitation area will be invited to take part in the observation study. Written informed consent will be obtained from nursing staff who agree to participate. The researcher will observe consenting emergency nurses working in the resuscitation area managing any adult (≥16 years old) critically ill patients triaged category 1 or 2.

#### Interview participants

Once observations have been completed and saturation reached, emergency nurses at the two observation sites will be invited to participate in a face-to-face interview. All emergency nurses with four or more years’ clinical experience of working in the resuscitation area and who have recently managed a critically ill patient triaged category 1 or 2 within the last six months will be eligible for participation. Written informed consent will be obtained from nursing staff who agree to participate.

### Data collection

There are three sources of data collection.

#### Phase 1: Survey

There is an absence of empirical data concerning nursing pain assessment and management of critically ill patients in the ED. Consequently, a Delphi technique will be used to develop survey items exploring emergency nurses’ practices in managing acute pain in critically ill patients. The Delphi technique is a method for achieving convergence of opinion concerning real-world knowledge solicited from experts within certain topic areas, and is in keeping with a mixed-methods framework [49]. The Delphi will consist of an expert panel of seven emergency nurse specialists from metropolitan and regional EDs. Studies employing the Delphi technique make use of individuals who have knowledge and experience of the topic being investigated [49]. As nursing role titles and descriptions vary across Australia, the following inclusion criteria will be applied: i) Registered Nurse; ii) currently working in an ED; iii) holds a postgraduate qualification in emergency nursing or greater; iv) has over five years clinical experience; and, v) is Clinical Nurse Consultant or Nurse Educator.

Consensus level needed to be reached following stability (<15% variation between rounds) [50] will be 85% (6 out of 7 panel members). It is anticipated that the Delphi study will consist of three rounds, with the preliminary question being: what are the core outcome variables to measure adequate management of acute pain in critically ill adult patients? The final survey will be administered through a secure Web-based platform. Emergency nurses working in a NSW ED who have contact with critically ill (ATS category 1 or 2) adult (≥16 years old) patients in the last six months will be invited to participate. Electronic reminders will be sent at 3 and 6 weeks after the initial invite. A link will be provided for participants to further disseminate the survey (i.e. snowballing) [51].

#### Phase 2: Observation

The second phase of data collection will comprise observations by the primary author (WV) as a non-participant observer in the resuscitation area of two NSW trauma designated EDs. Observations will be recorded as field notes to capture understandings of how emergency nurses detect, assess and manage acute pain in critically ill patients in everyday practice [52]. There are a lack of studies using observation to identify nurse-initiated analgesia, its safety and impact on patient outcomes; most data collection techniques have included survey/questionnaire [35, 39, 53], interview [37, 54, 55] or audits [21, 24, 36, 37, 39, 56–58]. These methods however reflect only reported, not observed actual practice. Undertaking observations of emergency nurses’ behaviours and interactions when delivering care to critically ill patients will add an outsider perspective to capture activities, processes, practice behaviours and context [59].

The non-participant observer (WV) will first introduce themselves to clinicians present in the resuscitation area. The study will be described in general terms as an observational study into how nurses manage critically ill patients. The specific details of data to be collected will be omitted to avoid unduly influencing nurse performance. Written informed consent will then be obtained. The researcher will then position themselves in such a way to observe but not obstruct emergency nurses or other members of the care team in their work. While openness is emphasised in qualitative inquiry, the observer will use an observation guide to assist field note recording of observations in such a complex sociotechnical setting [60] (Table 2).

**Table 2:** Observation guide

Observation dimension	Description
Space	The positioning of the resuscitation area in relation to the department, the overall physical layout of the resuscitation area and bed space
Activity	Movement, interaction or a set of interrelated actions that occur between emergency nurses and/or other healthcare providers
Act	A single action undertaken by an emergency nurse or care team member
Time	A particular point, period in time, pace or order of event that occur
Actor	Range of healthcare clinicians
Object	The type, arrangement of physical things that are present
Event	Activities that emergency nurses carry out, respond to
Goal	Things that emergency nurses set to accomplish

Observation sessions will continue until data saturation has been achieved and observed instances become repetitive [59]. Up to four weeks of observation (80–100 h) will be conducted in order to observe emergency nurses (*n* = 8–15) and critically ill patients (*n* = 18–25). This proposed period of time will allow sufficient opportunity to observe a wide variety of critically ill patients being managed in the resuscitation area, build trust and enable the observer to learn and understand the way emergency nurses’ practice in the everyday world [61].

#### Phase 3: Semi-structured interviews

In Phase 3, in-depth, semi-structured face-to-face interviews will be conducted by the lead researcher (WV) with a sample of emergency nurses (*n* = 10 to15) from the observation study sites. The purpose of face-to-face interviews will be to explore clinical experiences, feelings, attitudes and opinions that cannot be observed or easily shared in a group setting, and identify tacit skills and complexities embedded in the practice of managing acute pain in the critically ill patient [62]. Thus, they are a complementary method and serve as an additional data source of information. Interviews have been chosen as the last study phase to exclude any potential influence on responses to the survey or behaviour during observations, by raising awareness of emergency nurses’ practices in managing acute pain in critically ill patients.

An interview schedule will be informed by the survey (Phase 1) and observation (Phase 2) findings, and extant literature. Interviews will enable clarification and insight into the experiences, practice, decision-making, barriers and facilitators in managing acute pain in critically ill adult patients in the ED. Participants will be interviewed in a private area close to the clinical area for their convenience. All interviews will be audio-recorded and transcribed soon after recording to support data immersion and understanding of what has been said [63].

### Data analysis and integration

#### Quantitative data analysis

Survey data will undergo exploratory analysis to describe and identify consistencies or inconsistencies that might impact on the validity of the data findings. Appropriate descriptive statistics will be used to summarise data pertaining to individual variables, considering each variable’s level of measurement and the observed distribution of its data. Data will be analysed using IBM SPSS Statistics® 24.0 [64], with a *p*-value of <0.05 considered significant. Data distribution will be assessed via Q-Q-Plot, histograms generated and frequencies and percentages calculated. Depending upon the distributional properties of the measures, appropriate tests and analyses will be applied (e.g. ANOVA, Mann-Whitney, chi-square testing, Pearson’s correlation coefficient).

#### Qualitative data analysis

Descriptive statistics will be used to summarise demographic data collected from participants. Observations and interview audio recordings will be transcribed into text files and then imported into NVivo™ [65] for data management. The analytic process will be supported by Braun and Clarke’s [66] analysis framework. First, the researcher will familiarise themselves with the data by comparing field notes and audio-recordings during transcription. Second, a two-step process will be undertaken to generate initial codes. Initially, textual data will be reduced into smaller units: groups of words, sentences or paragraphs that contained particular aspects related to explore the research question. Each data segment will then be coded according to the essence identified from the unit of the data. Third, the researcher will cluster the codes to begin pattern generation and theme development. Theme development will be informed by the patterns and categories. After codes are categorised, the research team will review each cluster of codes to confirm patterns and meaning that accurately connect and express the data and thereby confirm themes [67]. Finally, themes and patterns pertaining to assessing and managing acute pain in critically ill adult patients by emergency nurses will be examined in relation to the underlying structure of Donabedian’s quality and safety model [45] and existing literature.

#### Mixed methods data integration and analysis

The integration and analysis of quantitative and qualitative data is what strengthens and gives richness to a mixed methods study [68]. Data integration and analysis within sequential explanatory mixed methods design occurs once all phases have been completed [69]. One of the most common purposes for mixing methods is complementarity, whereby quantitative and qualitative methods are used in a complementary fashion to answer related questions for the purpose of gaining a broader and more comprehensive understanding of the phenomenon [70]. Data transformation will further assist with quantitative and qualitative data integration [68]. In this study, quantitative data will first be presented in tables and histograms, with qualitative data displayed using charts (i.e. joint display) [71]. Second, quantitative data will then be qualitized through modal profiling [72], with narratives generated around the most frequently (i.e. modal) reported characteristics and practices in providing acute pain relief to critically ill patients. Third, qualitative data will be quantitized by transforming qualitative codes into numeric counts and variables [73]. Following data transformation, the transformed study data will be combined into a new data set. Lastly, to obtain a comprehensive description of the phenomena under investigation, the research team will then compare and contrast the quantitative data with quantitized qualitative data, and the qualitative data with the qualitized quantitative data; producing blended variables and meta-inferences [74]. To enhance the study rigour and validity of mixed methods research findings, the unified validation framework [75] for mixed methods research will be used. Consideration of strategies to meet criteria for validity is integral to building an optimal mixed-methods study design. A number of strategies [68, 76–78] have been described (Table 3).

**Table 3:** Unified Validation Framework, strategies to improve validity of mixed-methods research

Component, *definition*	Strategies
Foundational element, *researchers’ knowledge of the phenomenon of interest, methodology*	Detailed critique and description of the surrounding literature [1, 7, 83, 84]
Design quality, *appropriateness of methods and data analysis techniques in answering the research question*	In-depth description and rationale for research design, methods, data analysis and integration choices with reference to the extant literature Piloting of survey, observation and interview guide to insure accuracy and feasibility (internal validity) Use of multiple data sources to increase depth of understanding (dependability, reliability) Use of eligibility criteria and purposive sampling to ensure information-rich participants and observations. Prolonged engagement with the field/participants to ensure depth of understanding, reduce Hawthorn effect and to build trust (internal validity) Auditing of transcripts to ensure accuracy (dependability, reliability) Detailed, thick descriptive data (e.g. direct quotes) to assist reader interpretation and understanding of context (transferability, external validity) Use of reflective diary to recall decisions made, thoughts, feelings, instincts and challenges (confirmability, objectivity)
Legitimation, *collection and integration of quantitative and qualitative data*	Participant-driven data collection Use of and detailed description of complementarity framework in integrating quantitative and qualitative data Peer review/research team triangulation – coding, interpretation and generation of inferences Audit trail of decision-making and rationale throughout data collection, analysis and integration processes (dependability, reliability)
Interpretive rigor, *whether the meta-inference adequately incorporates inferences stemming from integrated data*	Peer review/research team driven generation of meta-inferences Audit trail of decision-making and rationale in generating of meta-inferences; data used, source(s) and weight within meta-inference
Inferential consistency, *relationship between findings and prior understandings, research and theory*	Detailed discussion of study findings and relationship to extant literature and theory - highlighting consistencies and discrepancies
Utilisation / Historical element, *how integrated data was used*	Audit trail of decision-making and rationale in selection of data used
Consequential element, *acceptability of findings, or inferences of a study*	Peer-reviewed publications Diverse range of participants Strengths, limitations and challenges described in detail

### Ethical considerations

All data will be collected, managed, analysed and stored in accordance with national ethical and scientific quality standards [79]. Participation in any part of this study will be voluntary; participants have the right to withdraw their consent and data at any time. All data will be de-identified prior to analysis. Phase 1 of this study protocol was approved by the South Eastern Sydney Human Research and Ethics Committee (17/162). Given the sequential nature of the study phases, with findings from one phase informing the development of the next, ethical approval will also be sought consecutively for each phase.

## Discussion

This will be the first comprehensive, integrated mixed-methods study to examine emergency nurses’ practices in assessing, monitoring and managing acute pain in critically ill patients. Emergency nurses undertake numerous clinical activities, often simultaneously or for multiple critically ill patients while working within the resuscitation area. The degree of knowledge and skills required to optimise and safely manage critically ill patients is highly complex, including acute pain management [7]. Adequate pain management is paramount in optimising comfort, pain relief and wellbeing of critically ill or injured patients. International guidelines concerning effective acute pain management recommend adequate assessment in all patients, with practice systems in place to ensure appropriate and timely analgesia assessment, and frequent monitoring and reassessment [11, 80, 81]. Emergency nurses are optimally placed to assess and initiate pain relief [24, 41], however the complexity of emergency nursing practice, judgment and factors influencing the detection, assessment and management of pain for critically patients is unknown. This study will provide answers to addressing a critical knowledge/practice gap in the science of emergency nursing, practice and literature regarding the assessment and management of pain in critically ill adult patients in ED.

The incorporation of Donabedian’s expanded quality and safety model [45] into the data collection and analysis, including the examination of factors influencing the clinical decisions and actions of emergency nurses, will develop a clearer understanding of what must be addressed to optimise acute pain management of critically ill patients in the ED. The systematic inquiry into the clinical environment (Antecedents) and model of care (Structure), the actions and interactions of emergency nurses in managing acute pain (Process), and, their influence in managing acute pain in critically ill or injured patients (Outcome), will assist this study to produce meaningful, practical and measurable recommendations concerning practice, education and policy.

### Strengths and limitations

This study has several strengths, including its robust and straightforward design. Using a sequential explanatory mixed methods study design will enable rich exploration of the existing practice of emergency nurses in assessing, monitoring and managing acute pain in critically ill adult patients. The use of quantitative and qualitative methods and integration of data will provide the research team with multiple perspectives from which to understand the complex and multidimensional nature of nursing practice and pain management in the ED.

There are several potential limitations that need to be considered. Sequential mixed methods studies are time-consuming to undertake, but it is felt that the richness of data obtained makes this an appropriate approach. Recruiting eligible survey participants will be engaged through email, web-based and other indirect methods of information dissemination. Consequently, emergency nurses who did not have reliable access to the Internet or membership to the College of Emergency Nursing Australasia may be excluded from participation. Strategies have been proposed to limit the impact of this and to increase survey response rate [51].

Observations and interviews will be undertaken in two trauma designated EDs, which may limit transferability of study findings. In undertaking observations and interviews in two large trauma designated ED facilities, it will increase the number and diversity in demographic characteristics of emergency nurses and critically ill patients; thereby developing a deeper understanding of how emergency nurses manage acute pain across a wide spectrum of critically ill patients, and increase transferability and generalisability of the study findings.

In observing emergency nurses within the clinical environment, nurses may demonstrate an increased awareness in assessing, monitoring and communicating with their patients about their comfort and therefore pain because of being observed. Strategies have been highlighted to address this issue. In additional, it has also been demonstrated previously that it is difficult for individuals to sustain behaviour that is dramatically different from normal for any length of time in a busy health care environment [82].

## Conclusion

This protocol outlines a multiphase sequential explanatory mixed methods study that will guide investigation of emergency nurses’ perceptions and practices in assessing and managing acute pain in critically ill adult patients. Outcomes of this study will provide urgently needed insight into knowledge gaps on how acute pain is managed in critically ill patients in the ED, how barriers are overcome and what resources are required to facilitate optimal patient care and safety. These findings will serve as a knowledge base to expand theory and inform research and practice in this important and evolving area of emergency practice.
